# Low-cost fabrication of flexible tactile sensor arrays

**DOI:** 10.1016/j.ohx.2022.e00372

**Published:** 2022-10-31

**Authors:** Niklas Fiedler, Philipp Ruppel, Yannick Jonetzko, Norman Hendrich, Jianwei Zhang

**Affiliations:** TAMS, Department of Informatics, Universität Hamburg, Germany

**Keywords:** Tactile sensing, Customizable sensor arrays, Wireless sensors, Flexible tactile sensors

## Abstract

While for vision and audio the same mass-produced units can be embedded in many different systems from smartphones to robots, tactile sensors have to be built in application-specific shapes and sizes. To use a commercially available tactile sensor, it can be necessary to develop the entire system around an existing sensor model. We present a set of open-source solutions for designing, manufacturing, reading and integrating custom application-specific tactile matrix sensors. Our manufacturing process only requires an off-the-shelf cutting plotter and widely available plastic and metal foils. This allows creating sensors of diverse sizes, shapes, and layouts, which can be adapted to various specific use cases as demonstrated with exemplary robot integrations. For interfacing and readout, we develop an Arduino-like prototype board (Tacduino) with amplifier circuits to ensure good resolution and to suppress crosstalk. As an example, we give step-by-step instructions to build tactile fingertips for the RobotiQ 3-Finger Gripper, and we provide design files for the readout circuit board together with Arduino firmware and driver software. Both, wired and wireless communication between the sensors and a host PC are supported by this system. The hardware was originally presented and investigated in [1].

## Graphical abstract

**Figure:**
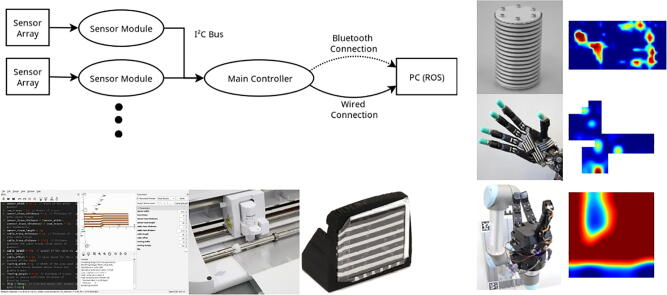

Specifications table:

**Table:** 

**Hardware name**	System of fully customizable tactile sensor arrays
**Subject area**	Robotics and Automation
**Hardware type**	Electrical engineering and computer science
**Closest commercial analog**	Closest to our work are the products of Tekscan, Inc. and the Taktilus Free Form product line from Sensor Products Inc..
**Open source license**	GPL 3
**Cost of hardware**	295 € (127 €without cutting plotter)
**Source file repository**	https://doi.org/10.17605/OSF.IO/JE8KN

## 1 Hardware in context

While vision remains the dominating modality in robotic perception, tactile sensing is also extensively researched for both humans and robots as shown in surveys of the field [2], [3]. Generally, tactile sensors for robotic setups can be divided into two groups: those which are designed for texture perception and the ones focusing on force measurements. The sensors of the former type are usually camera-based, meaning that a camera is taking an image of a surface or a membrane that makes contact with the stimulating object. This results in a high spatial resolution but inaccurate force measurements. A promising recent implementation of such sensors is the DIGIT sensor [4]. Its design files are open source, but the assembled sensor is also commercially available [5].

In this work, however, we focus on the latter group, i.e. sensors designed for precise force perception with a lower spatial resolution. Most sensors of this type work either with resistive [6] or capacitive cells called taxels (a combination of the words “tactile” and “pixel”). Various versions of such skin-like sensors are commercially available, but the problem with using them lies in integration. Only fixed sizes and configurations are available. The choice in sensor layout, taxel distances, and sensitivities is very limited. When integrating these sensors into a robotic system, many spatial and sensing requirements need to be met because the existing and commonly mechanically complex robot hardware needs to be accommodated [7], [8]. Thus, there are many situations in which existing commercial products cannot be applied.

We explain the fabrication of the sensor system in Section 5 at the example of the Robotiq 3-Finger Gripper [9]. Its three fingers with three joints each are underactuated as only a single motor moves each finger. A mechanism of springs and levers enables various types of grasps, but without a tactile sensor, very little about the type and the success of a grasp is known. The MEMS-based sensors first presented by Tenzer et al. in 2014 use barometric sensors cast into silicone in a vacuum for tactile sensing. The sensors themselves and a specialized kit for the Robotiq gripper were commercially available for a while [10]. Their minimum cell size of $6\times 6\;{\mathrm{mm}}^{2}$ yielded a relatively low spatial resolution (we achieve a minimal cell size of $2\times 2\;{\mathrm{mm}}^{2}$ with our sensors), but they still found usage in research applications [11]. Independently from each other, researchers from the ETS Montreal and the Stanford Biomimetics and Dexterous Manipulation Lab (BDML) also worked on the development of tactile sensor systems specifically for the Robotiq 3-Finger Gripper. Both sensors are capacitive and require advanced production techniques. The Montreal team presented and evaluated their sensor system in [12]. A different sensor was presented one year later, in 2017, by the same team on a Robotiq 2-Finger Gripper [13]. The approach of the team at Stanford implements a version of a design proposed by Ulmen et al. in 2010 [14] specifically tailored for the gripper.

The Willow Garage PR2 robot is equipped from the factory with tactile sensor arrays integrated into the tips of its two-finger grippers. For example, it was used by Chitta et al. in their work about manipulating and investigating objects [15].

While these sensors yield very impressive results, they are not commercially available at this moment and are very hard to replicate. Especially for researchers who do not focus on sensor development and production, building such sensors is not an option. Because of their simplicity, resistive tactile sensors find applications in many self-built approaches with diverse configurations depending on the requirements of the given applications [16], [17], [18]. In general, related works and commercial products often focus only on the sensor or sensor array itself but not the whole system and robot integration. Also, they are often not sufficiently customizable to be integrated into existing robotic systems. We approach the issues of system integration, adaptability in resolution, size and shape, and flexibility with the sensor arrays presented in this work.

## 2 Hardware description

**Fig. 1:**
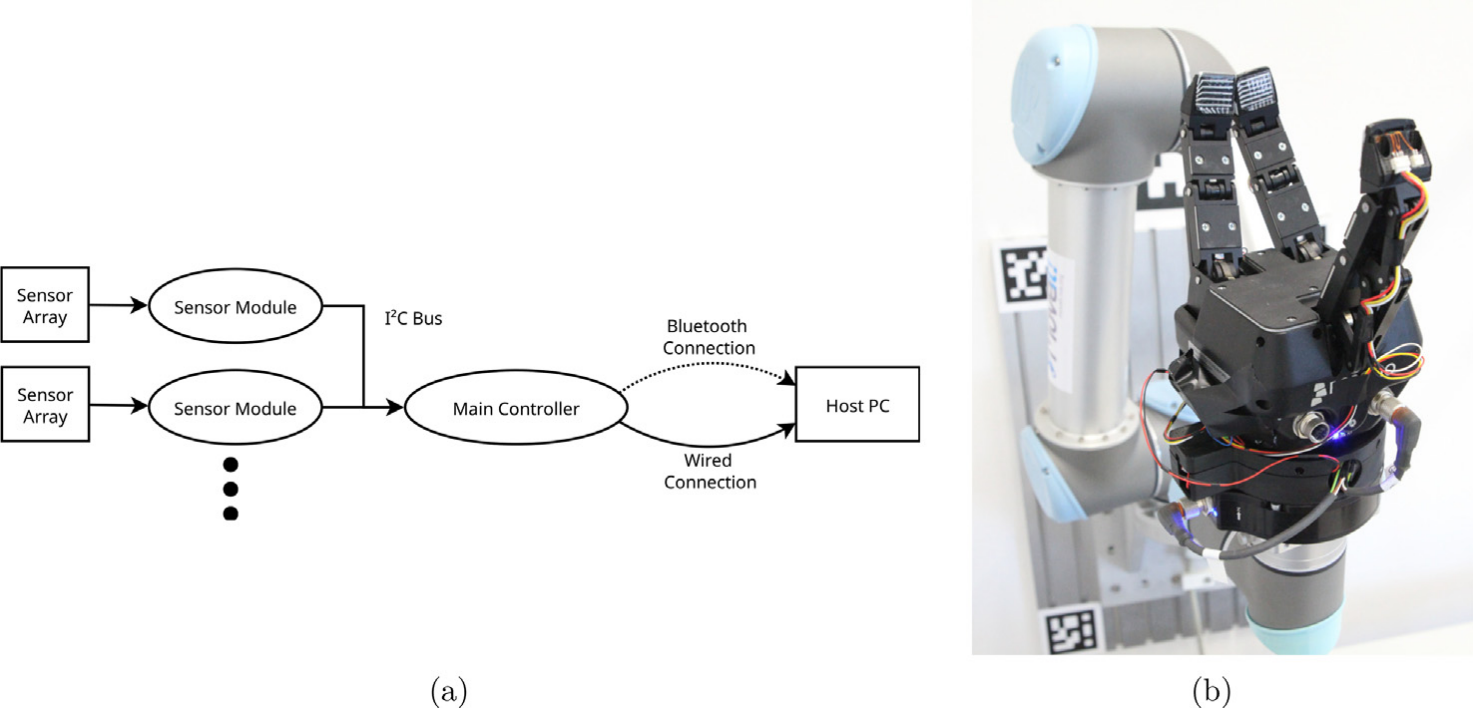
(a) A schematic depiction of the proposed system. Each sensor array is attached to a dedicated sensor module. The sensor module reads the data from the sensor array and makes it available to the I^2^C bus. The main module is used as the master of the bus and pulls the measurements from each sensor module. After pulling the data from one module, it is transferred via Bluetooth or a wired connection to the host PC. (Modified version of a figure in [1].) (b) A system consisting of three sensors and sensor modules (inside the fingertips) and one main module with a Bluetooth adapter (on top of the gripper, wrapped in tape) integrated onto a Robotiq 3-Finger Gripper.

A schematic description of our system is shown in Fig. 1a. Highly customizable tactile sensor arrays are each directly connected to a sensor module. The sensor module combines the electronics to amplify and decouple the sensor signals with a microcontroller reading the analog sensor values and providing them to the I^2^C bus. A main module acts as the master of the bus and sequentially requests sensor readings from each sensor in the module. After receiving tactile data from a sensor module, it is transmitted to the host PC via a USB or Bluetooth connection.

### 2.1 Sensor array

**Fig. 2:**
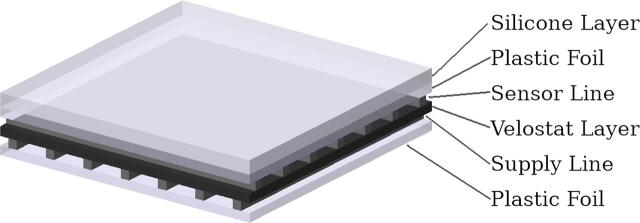
Schematic representation of the sensor and its layers [1].

The sensor arrays used in the system are designed to be easily reproducible resistance-based pressure sensors utilizing the conducting properties of piezoresistive foil. Velostat foil is originally intended as a packaging material for electronic components which are sensitive to electrostatic discharge (ESD). However, its contact resistance decreases in relation to the contact force. This was analyzed in detail by Dzedzickis et al. [19]. We make use of this by placing aluminum traces at a 90° angle to each other on both sides of one layer of the piezoresistive Velostat foil. Fig. 2 depicts a schematic view of the sensor layers. The Velostat layer is sandwiched between two layers of aluminum traces (Sensor Line and Supply Line). These traces are glued to a carrier foil (Plastic Foil). Optionally, a layer of silicone is placed on top of the sensor to adapt its characteristics. A layer of silicone spreads the force of the stimulus over a greater sensor area. This slightly lessens the sensor’s sensitivity, but also reduces the effect of blind spots between taxels because their sensing area grows.

### 2.2 Sensor module

**Fig. 3:**
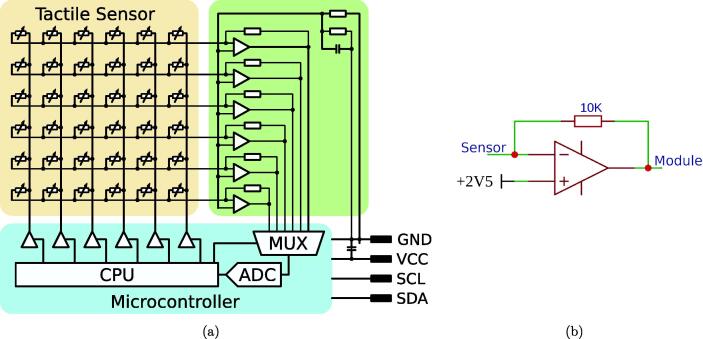
(a) Exemplary schematic of a 6×6 sensor array (orange) with a microcontroller (blue), transimpedance amplifiers, and an I^2^C data bus [1]. (b) The transimpedance amplifier used for amplifying and decoupling sensor readings.

**Fig. 4:**
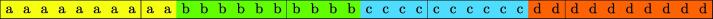
Exemplary distribution of four 10-bit values (a, b, c, and d) into five 8-bit values.

**Fig. 16:**
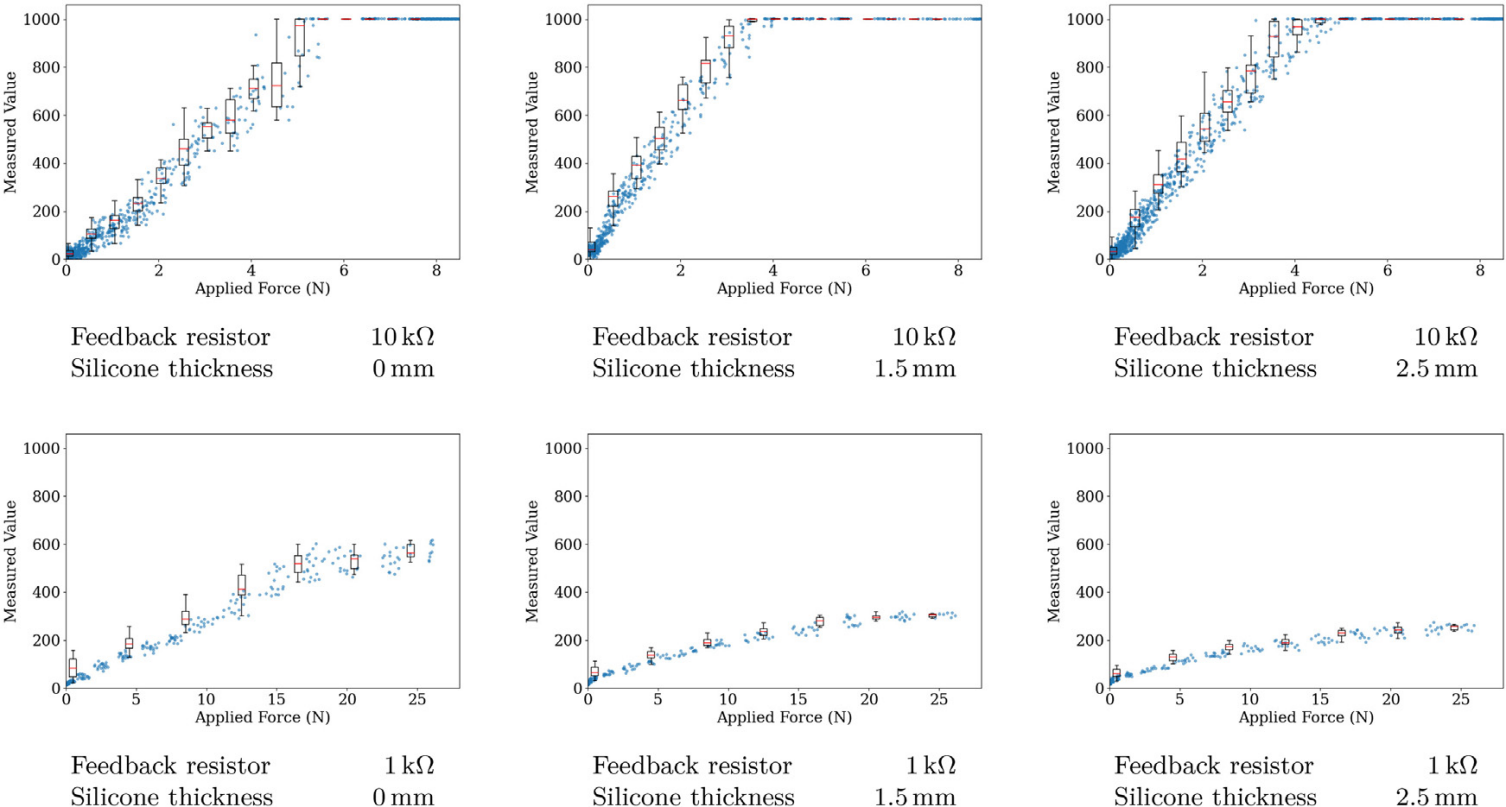
Sensor stimuli and measurement values plotted against each other with various sensor configurations regarding the feedback resistor value and the silicone layer thickness.

The sensor module is tasked with reading out the tactile sensor, decoupling its measurements, and making the acquired data available via the I^2^C bus. This is implemented with a microcontroller and a series of transimpedance amplifiers. A schematic of a sensor module attached to a sensor is shown in Fig. 3a. The sensor is modeled as an array of variable resistors. The microcontroller (Fig. 3a, blue) then applies 5 V to one of the sensor supply lines (vertical in the figure). Afterwards, the output voltages of the transimpedance amplifiers (to the right of the sensor and in more detail in Fig. 3b) are read by the analog to digital converter (ADC) of the microcontroller. Crosstalk between the taxels of the sensor is suppressed by the transimpedance amplifiers, which pull all sensor lines (horizontal in the figure) to the same reference voltage. The feedback resistor of the amplifiers regulates the sensor’s sensitivity. We are using 10kΩ feedback resistors in our sensor boards. A lower value of the feedback resistor reduces the sensitivity and causes later saturation, as shown in Fig. 16. The microcontroller compresses the information read from the sensor following a pattern shown in Fig. 4 and caches it. When the main module requests the sensor data via the I^2^C bus, the sensor module will respond with the most recently cached sensor reading. Thus, the sampling rate of the sensor is also dependent on its resolution. In some use cases, a reduced resolution might be beneficial to warrant a higher sampling rate.

**Fig. 5:**
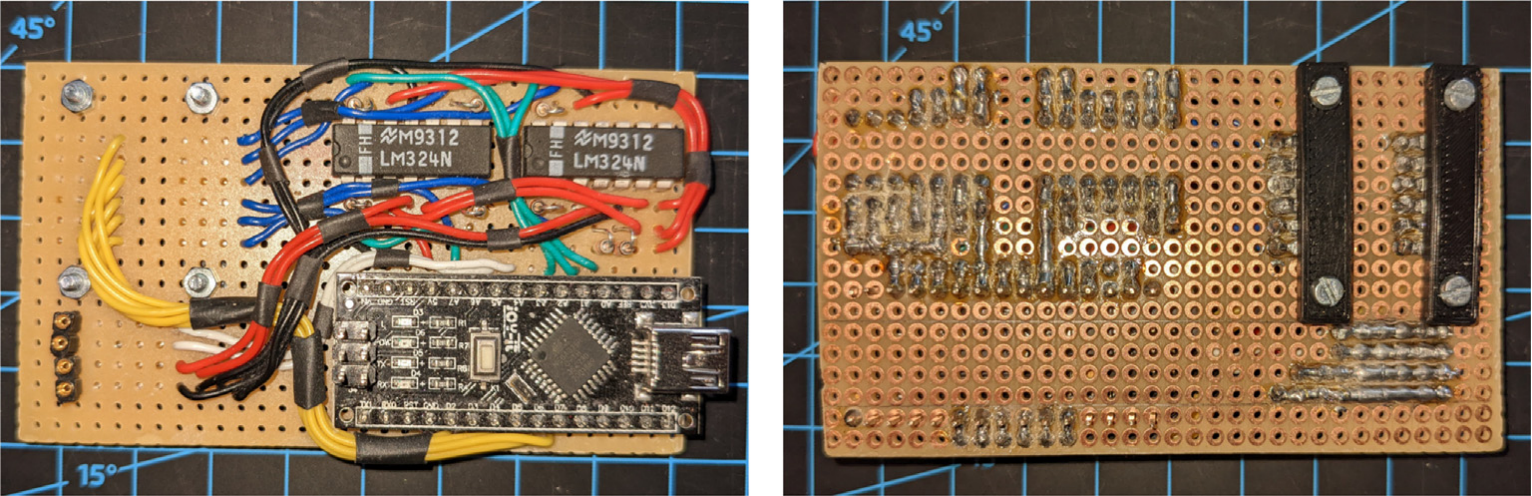
Prototype of the sensor module on a perfboard from the front (left) and back (right).

An early perfboard implementation of a sensor module prototype is shown in Fig. 5. It utilizes an off-the-shelf Arduino Nano clone and two LM324N in a DIP-package. Each of the LM324N includes four op-amps, but altogether only six are used as the Arduino Nano only has six analog channels available. Two of the eight ADC pins are used for the I^2^C bus connections. Red and black cables handle the VCC and ground connections, respectively. The connections between the sensor lines and the transimpedance amplifiers are blue. Yellow cables are the sensor supply lines directly from the Arduino to the sensor traces. The cables connecting the amplifiers to the analog inputs of the Arduino are green. The data lines used for SDA and SCL of the I^2^C bus are colored white.

After we successfully tested the prototype, we designed a custom printed circuit board (PCB) for a sensor module with a very small footprint to be universally applicable. We wanted it to still be easy to solder by hand, so TSSOP and TQFP packages were used in the design of the boards instead of ball grid arrays or other no-lead packages such as TDFN. While this increased the footprint of the board slightly, it allows for a significantly easier building process.

One of the greatest mechanical challenges of the project lies in establishing a stable connection between the sensor traces and the sensor module. For our prototype (see Fig. 5 and a debugging setup, we used a clamping mechanism that presses the sensor cable traces onto the perfboard. However, this implementation is not applicable with the tight space restrictions we faced when integrating it into a robot setup. In such situations, we use conductive tape and a lower clamping force, as further explained in Section 5.3.

### 2.3 Main module

**Fig. 6:**
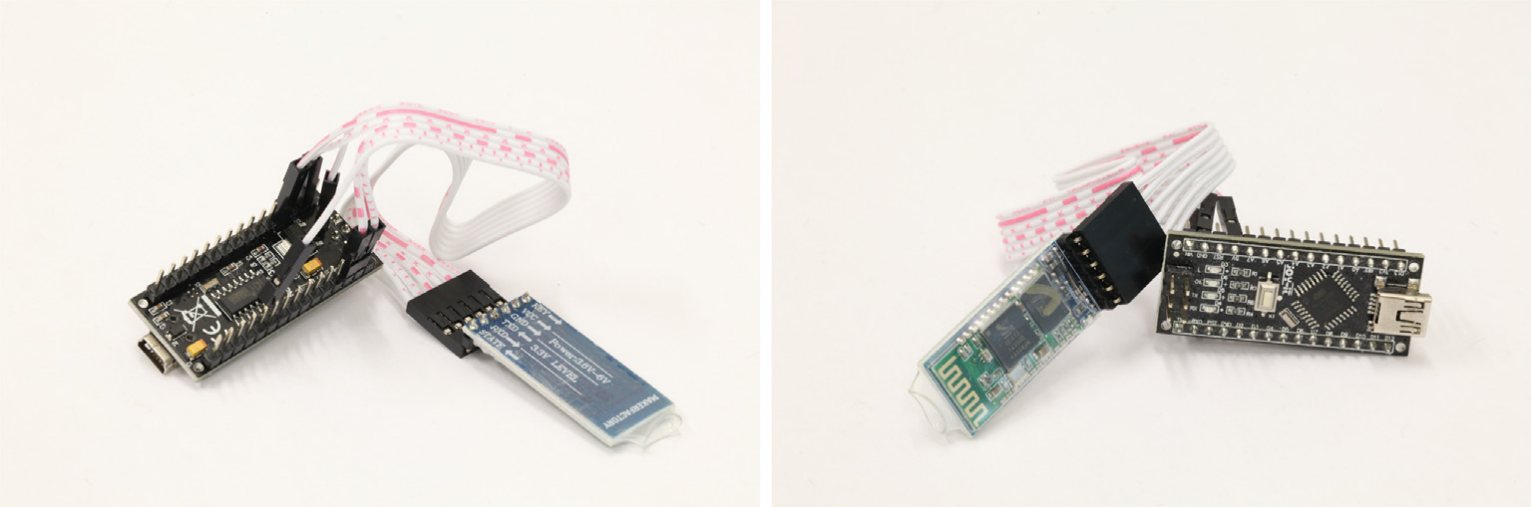
Simple implementation of the main module.

In our setup, an Arduino Nano clone is used as main module. It acts as a master on the I^2^C bus and sequentially pulls measurements from the sensor modules registered to it. When receiving data from one of the sensor modules, the data is transmitted to the host PC. For data transmission, a wired, as well as a Bluetooth connection, are supported. While the wired connection supports higher frame rates and lower latencies of the measurement data, a wireless connection offers more freedom of movement when integrated into a robot. The Bluetooth connection is implemented by attaching an HC-05 Bluetooth module to the Arduino. In our case, there were no tight space restrictions for the main module because it can be placed anywhere on the robot requiring only four wires to connect to (GND, VCC, SDA, and SCL). Thus, we did not design a custom PCB allowing us to rely on proven parts without the risk of design errors. Our setup is shown in Fig. 6. Theoretically, other devices such as a Raspberry Pi Pico, ESP32, or an Arduino Nano 33 IoT could be used to allow more I^2^C channels or WIFI data transmission.

### 2.4 Host PC

Any machine running a current version of Linux and optionally ROS can be used as the host PC. Our setup runs Ubuntu 18.04 and ROS melodic. Bluetooth is supported by some mainboards and can otherwise be retrofitted by plugging in a generic USB to Bluetooth adapter. The driver scripts are written in Python and read the compressed data transmitted from the main module, unpack it and make it available either for a Python script or ROS nodes. A configuration file is used to set all parameters of the system and the connection for the data transfer. Section 6 provides further details about the configuration of the system.

The system presented offers multiple advantages over existing approaches:

• Low cost in both material and tools required
• Relatively easy construction process for non-experts
• Fully customizable sensor layout and sensitivity
• Wireless data transmission capability
• Bendable sensors
• Modular design

## 3 Design files summary

.

**Table:** 

**Design filename**	**File type**	**Open source license**	**Location of the file**		
PCB_Sensor Module V1.1.json	PCB layout	GPL 3	https://doi.org/10.17605/OSF.IO/JE8KN		
traces.scad	CAD file	GPL 3	https://doi.org/10.17605/OSF.IO/JE8KN		
traces_a.svg	Vector graphic	GPL 3	https://doi.org/10.17605/OSF.IO/JE8KN		
traces_b.svg	Vector graphic	GPL 3	https://doi.org/10.17605/OSF.IO/JE8KN		
robotiq_3_finger_gripper_tactile_ sensor_adapter.FCStd	CAD file	GPL 3	https://doi.org/10.17605/OSF.IO/JE8KN		
robotiq_3_finger_gripper_tactile_ sensor_adapter_in.stl	CAD file	GPL 3	https://doi.org/10.17605/OSF.IO/JE8KN		
robotiq_3_finger_gripper_tactile_ sensor_adapter_out.stl	CAD file	GPL 3	https://doi.org/10.17605/OSF.IO/JE8KN		

The sensor module is a custom-designed PCB to fit our requirements. It was required to be small enough to fit into our fingertip adapter (robotiq_3_finger_gripper_tactile_sensor_adapter.FCStd) for the Robotiq 3-Finger Gripper as described in 5.1. PCB_Sensor Module V1.1.json is an EasyEDA file. Using the tool hosted at https://easyeda.com/, the file can be uploaded, customized, and the necessary production files (Gerber, BOM, Pick and Place) to order it from https://jlcpcb.com/ can be generated. In our case, one side of the PCB will be assembled by the manufacturer, the other side needs to be assembled by hand. Still, a full assembly is possible with different manufacturers, and soldering all parts by hand is also an option. With traces.scad custom traces can be designed easily in OpenSCAD to generate SVG files for the cutting paths. traces_a.svg and traces_b.svg are the cutting paths we used for our sensor built as described in Section 5.2.

## 4 Bill of materials summary

.

**Table:** 

**Vendor number**	**Component**	**Quantity**	**Cost per unit – currency**	**Total cost – currency**	**Source of materials**	**Material type**	
PORTRAIT 3	Silhouette Portrait 3 Plotter	1	168.03 €	168.03 €	reichelt.de	Non-specific	
1678142	Arduino Nano clone	1	13.33 €	13.33 €	conrad.com	Non-specific	
Custom	Sensor module PCB (one side assembled)	5	6.04 €	30.20 €	jlcpcb.com	Non-specific	
556-ATMEGA328P-AU	8-bit Microcontroller	1	2.22 €	2.22 €	eu.mouser.com	Semi-conductor	
603-CC603KRX7R9BB104	Ceramic capacitor 0.1uF	3	0.08 €	0.24 €	eu.mouser.com	Ceramic	
903503571203	Aluminum adhesive tape RK-140, 50 mm, with carrier foil	1	13.51 €	13.51 €	esska.de	Metal	
541274	Aluminium tape, Silver, 50 mm, without carrier foil	1	9.16 €	9.16 €	conrad.com	Metal	
540923	Packaging tape, transparent, 50 mm	1	5.83 €	5.83 €	conrad.com	Composite	
TESA 56170	Double-sided adhesive tape, 50 mm	1	2.10 €	2.10 €	reichelt.de	Composite	
NOPI 55513	Crepe tape	1	4.66 €	4.66 €	reichelt.de	Other	
1656	Z-Axis conductive tape	1	4.95 $	4.95 $	adafruit.com	Other	
EXP-R15-355	Pressure-sensitive conductive sheet (Velostat)	1	5.25€	5.25€	exp-tech.de	Composite	
4721489	Oven bag	1	1.67 €	1.67 €	shop.rewe.de	Composite	
3379994747954	10 JST 1.5 mm ZH 4-Pin cables	3	10.08 €	30.24 €	amazon.de	Non-specific	
SIL BLADE-AUTO-2	Silhouette cutting blade	$2^{★}$	12.56 €	25.12 €	reichelt.de	Non-specific	
3379994748227	100 JST 1.5 mm ZH 2-Pin cables	$1^{★}$	10.92 €	10.92 €	amazon.de	Non-specific	
0227329	Silicone sheet, translucent, colorless, 1 mm	$1^{★}$	6.65 €	6.65 €	modulor.de	Composite	
2134049	HC-05 Bluetooth module	$1^{★}$	11.27 €	11.27 €	conrad.com	Non-specific	

At least one sensor can be produced with the specified amount of materials. The total cost is approximately 295  € (without tax), but it should be noted that the plotter must be purchased only once and the more sensors are produced, the cheaper it gets. For some parts (custom PCB, cables), there is a minimum order quantity. All parts marked with a star are optional and not necessary for a working sensor.

### 4.1 Tools

**Fig. 7:**
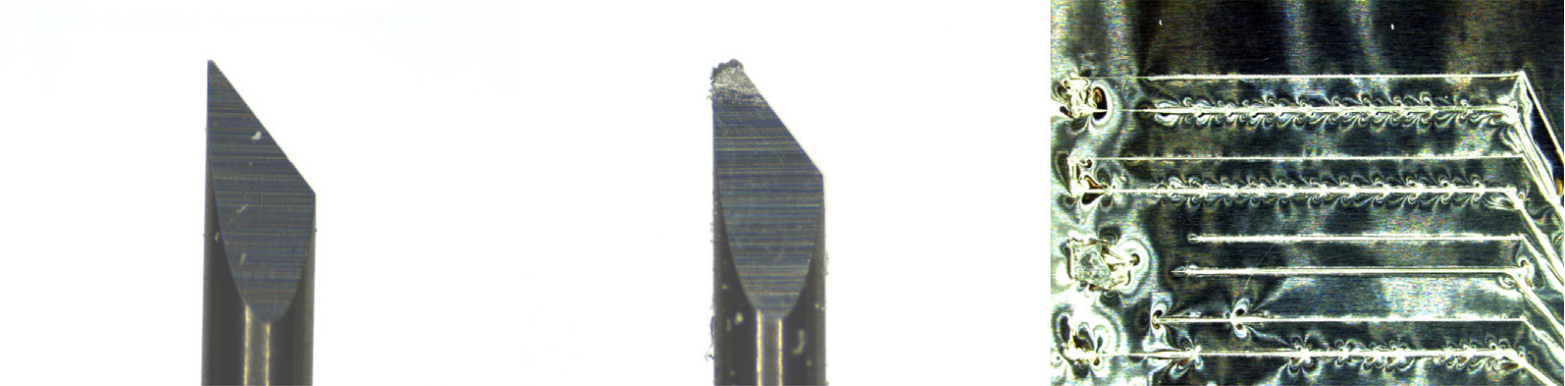
Micrographs of a new blade (left), a heavily used one (middle), and traces cut with a damaged blade (right).

Besides tools needed to solder the PCBs, a cutting plotter is required. In our case, the hobby-grade cutting plotter Silhouette Portrait 3 proved to be cost-effective and sufficiently precise. Note that a suitable cutting area is required and that this area includes the cable traces leading to the sensor. One axis is limited by the width of the aluminum tape and the other by the plotter. Regarding the cutting blade of the plotter, we recommend ordering multiple replacements because they can break easily during usage as they are not designed for cutting aluminum tape. Fig. 7 shows a comparison between a new blade and a broken one under a microscope. It is evident that the tip of the blade broke, and the remainder of it is significantly damaged. The rightmost picture in the figure shows cutting errors due to a broken blade. The traces are not cut reliably anymore and are wrinkled. The wrinkles can negatively impact measurement precision as compared to flat surfaces, since they interact differently with the pressure-sensitive foil.

### 4.2 Tapes

We utilize two types of aluminum tape in this work. One roll with a carrier foil and another one without. The tape without a carrier foil is coated to allow the glue of the tape to detach from the roll. However, this coating also prevents crepe tape from reliably sticking to it during the fabrication process of the sensor. Thus, it is not possible to transfer the traces onto a carrier foil after cutting. This problem does not occur with the tape with a carrier foil as it is not coated. Still, we leverage the properties of the coated tape by using it as the cutting surface. The aluminum tape to be cut sticks well enough to it not to detach during the cutting process but can still be pulled off for the transfer onto a carrier foil. The process of cutting the sensor is explained in detail in Section 5.2.

## 5 Build instructions

Before starting with sensor production, a draft of the overall sensor system is required. This means that the number of sensors (and their sensor modules) needs to be determined. Based on that number and the frame rate required, it needs to be decided how many main modules are used and which sensor modules are connected to which main modules. To show the fabrication process, we design and produce a fingertip sensor for the Robotiq 3-Finger Gripper with a 6×6 tactile array.

### 5.1 Sensor design

A sensor design consists of the traces of two electrode layers making up the sensor lines, supply lines, and cables of the sensor. During the sensor design, it is important to consider the feasibility of the production and the size of the overlap between the supply and sensor traces. Regarding the width of sensor traces, we recommend using a minimum of 1 mm, as otherwise, the aluminum tape will detach from the carrier foil during cutting, which can result in damage to the traces. Therefore, we suggest a minimal taxel size of 1mm×1mm with a minimum distance of 1 mm between taxels. Thus, an array with 6×6 taxels can be designed to be as small as 11mm×11mm. An inconsistent overlap between supply and sensor traces can result in a heterogeneous pressure sensitivity across the sensor array.

**Fig. 8:**
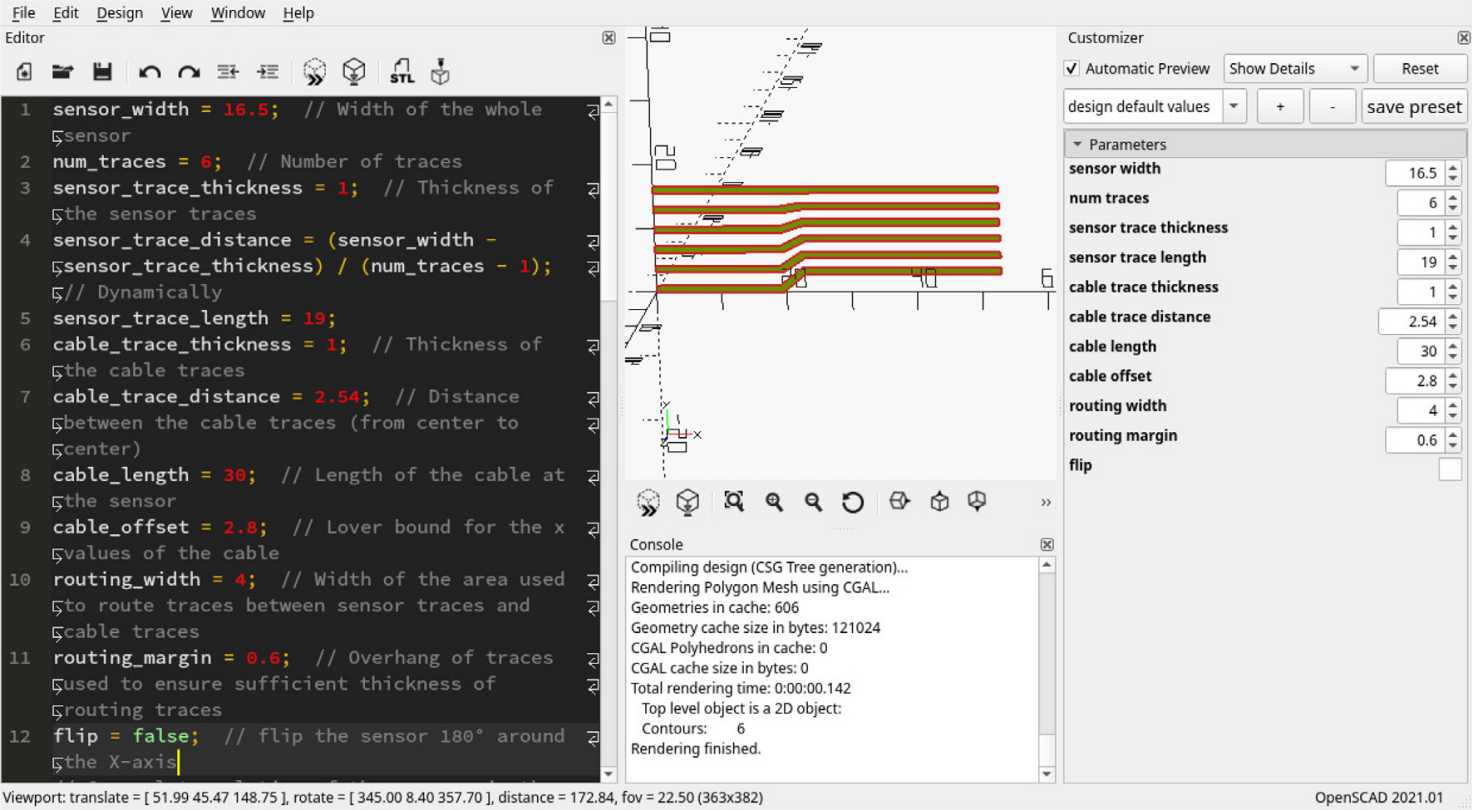
Parameterized sensor trace design file opened in OpenSCAD. Various parameters can be easily changed with direct visual feedback.

**Fig. 12:**
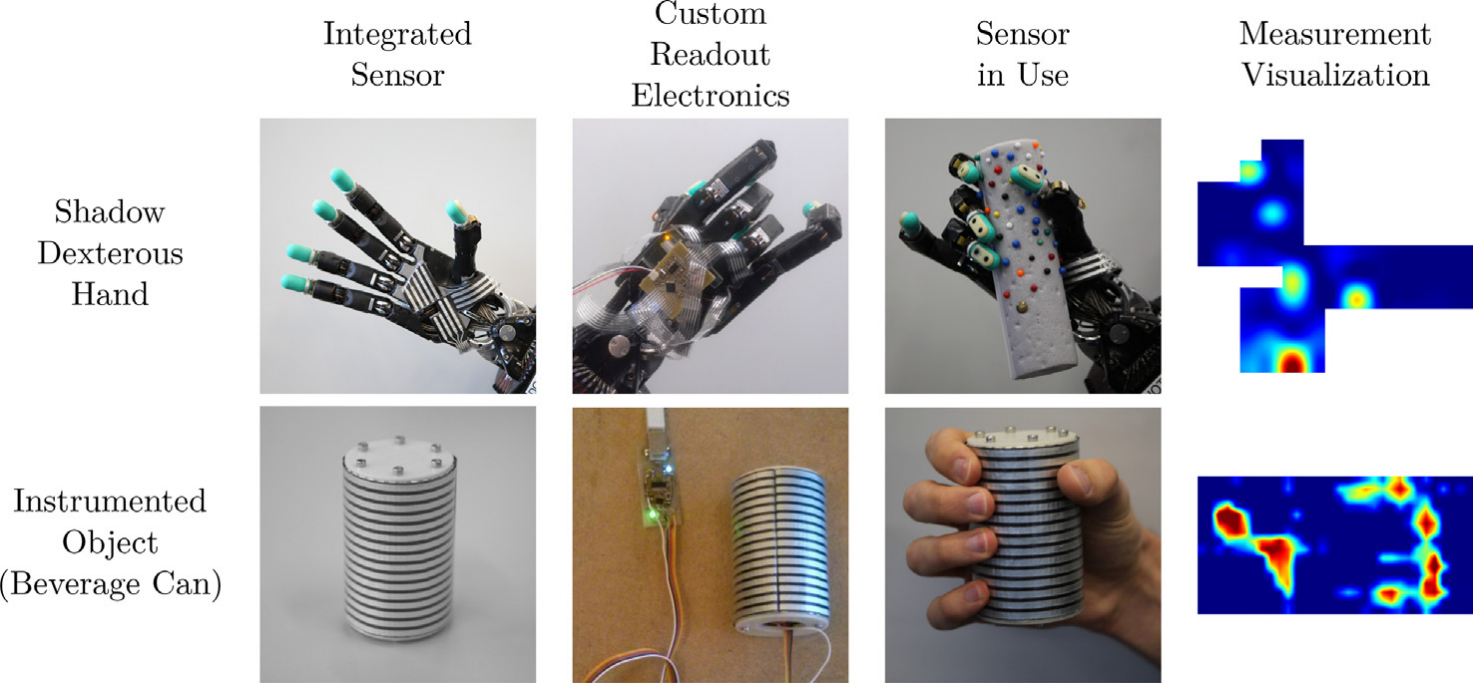
Our sensor system integrated into a Shadow Dexterous Hand and an instrumented object with customized sensor layouts, readout electronics, and visualizations of their reaction to given stimuli. (Images partially from [1].)

Instead of the software delivered with the cutting plotter, we are using a pipeline composed of OpenSCAD, Inkscape, and an Inkscape plugin (https://github.com/fablabnbg/inkscape-silhouette) to create the design files and interface with the plotter. OpenSCAD is used to design the sensor traces and create an SVG file. Afterward, the file is imported into a new Inkscape DIN A4 template. There, it is placed in a position where it can be cut by the plotter. By selecting ”Extensions 〉 Export 〉  Send to Silhouette…”, a dialog will open up to set the plotting parameters and start the plotting process. The parameters we employed during plotting are listed in Table 1. We assume that similar parameters can be applied when the same plotter is used with a relatively new blade. The speed, pressure and blade depth should be chosen as low as possible to minimize wear on the blade. While the sensors are highly customizable (see Fig. 12), in most applications, they are rectangular. In our design files, we provide an OpenSCAD file describing a highly parameterized version of the sensor traces for rectangular sensors (see Fig. 8).

**Table 1:** Plotting parameters.

Parameter	Value
Cutting Mat	None
Speed	1
Pressure	33
Blade Depth	3
Repeat each stroke	2

### 5.2 Sensor production

As shown in Fig. 2, the sensor is composed of multiple layers, including the aluminum traces for the sensor and supply lines. Table 2 gives a detailed overview of the sensor production steps. (See Table 3.)

**Table 2:** Sensor production steps

		
Prepare the cutting mat of the plotter by first applying the coated aluminum tape (from the roll without the carrier foil). This provides a cutting surface to which the tape to be cut can stick reliably but can also be removed later on to transfer the traces to a permanent carrier foil. Then, apply a piece of the uncoated aluminum tape (from the roll with carrier foil) to be cut. Only the uncoated tape can reliably be transferred **(a)**. Insert the cutting mat into the plotter and start the plotting process **(b)**. We described the recommended process for our plotter in Section 5.1. Then, remove the plotted traces from the plotter **(c)**.		
		
Use tweezers and a scalpel if necessary to carefully remove the excess material of the aluminum tape (everything that is not part of the traces) **(d)**. Afterward, only the traces should be left on the prepared cutting mat **(e)**. Apply crepe tape as transfer foil for the sensor traces. Make sure that the crepe tape is pressed firmly on all parts of the traces **(f)**.		
		
Prepare the oven bag foil by cutting out a piece and taping it evenly on a flat surface **(g)**. Carefully remove the crepe tape carrying the traces from the cutting mat **(h)** and apply it to the oven bag foil **(i)**. Use firm pressure on the sensor traces to ensure good adherence between the traces and the oven bag foil and remove minor kinks in the traces. Note that kinks in the traces of the sensor area can result in erroneous measurements. After the traces were transferred, cut off the excess oven bag foil. Steps **(a)** to **(i)** need to be performed for both the sensor and the supply traces. Depending on the sensor size and the workspace of the plotter, it is possible to plot both layers at the same time.		
		
		
The final assembly of the sensor starts by fixing a strip of transparent adhesive tape with the sticky side facing up to a flat surface, similar to the oven foil in step **(g)**, using crepe tape **(j)**. Place the lower lines of the sensor with the aluminum traces pointing up onto the adhesive tape **(k)**. There, only the sensor area of the traces needs to make contact with the tape and not necessarily the cable part. Also, placing the cable part on the tape is possible and yields a stiffer cable. Again, make sure that there are no kinks in the tape and the traces. Cut a piece of Velostat foil to size and place it on the sensor traces **(l)**. Place the upper sensor lines with the aluminum traces pointing downwards onto the Velostat foil **(m)**. Ensure to stay clear of the adhesive tape with the cable part of the traces because the tape would insulate the cables and might break them during later removal. We use crepe tape to shield the sensor cables from the adhesive tape and to fixate the assembly **(n)**. Afterward, a strip of adhesive tape is applied from the top, conjoining all the sensor layers **(o)**.		
		
After removing excess material of the adhesive tape **(p)**, double-sided tape is applied to the back of the sensor **(q)**. Then, the sensor can be glued to its intended mounting position **(r)**. Optionally, another piece of double-sided tape may be used to attach a layer of silicone mat onto the sensor area to adapt the sensor characteristics.

**Table 3:** Sensor Module Integration Steps.

			
To flash the firmware onto the sensor modules, a programmer and a power supply need to be connected to it. For both, we use an Arduino Nano clone flashed with the Arduino ISP sketch. It is connected via two four-pin JST 1.5 mm plugs connected to solderable sockets. We can establish a connection to the board without soldering by pressing the pins of the sockets into the PCB **(a)**. In the Arduino IDE, set the board to ”Arduino Nano” and the processor to ”ATmega328p”. Then, burn the Arduino bootloader by selecting ”Burn Bootloader”. Afterward, open the sensor module firmware sketch. There, a suitable value for i2c_address needs to be set (in the range of 1 to 255). If a different array layout is used, enter the used digital pins (a subset of {4, 5, 6, 7, 8, 9}) and analog pins (a subset of {A0, A1, A2, A3, A6, A7}). Note that each address is only usable once per main module as the sensors are individually identified by that number. When the ID is set, upload the sketch by clicking ”Upload Using Programmer”. Separate the modules from each other **(b)**. If necessary, cut off the part of the PCB populated only with sockets and a status LED to save space **(c)**.			
			
In case the sockets were cut off, attach enameled wires to the solder spots on the PCB and to the sockets **(d)**. We use electrically conductive tape to attach the sensor wires to the sensor module, as a high mechanical pressure between the parts could not be ensured in this setup. We also evaluated the usage of various conductive inks, but they yielded significantly worse results. Cut a thin strip of the conductive tape **(e)** and apply it to both sensor connection areas of the PCB **(f)**. The tape only conducts in Z-direction and not along its width and length. Thus, a single piece can be used across multiple contacts.			
			
Trim the sensor cable traces to fit onto the sensor module. Note that it might be necessary (as for the lower cable in our build) that the sensor cable is folded back for the cable traces to make contact with the PCB **(g)**. Insert the sensor module into the fingertip case and bend the cable traces onto the PCB. Make sure that the cable traces align with the pads on the PCB. Also, prevent cable traces from making contact with other parts such as the capacitors C4, C5, and C6 **(h)**. Finally, close the fingertip housing by seating the inner part and routing the cables outside **(i)**. The components are held together by the mounting screws of the fingertip itself.			

**Fig. 9:**
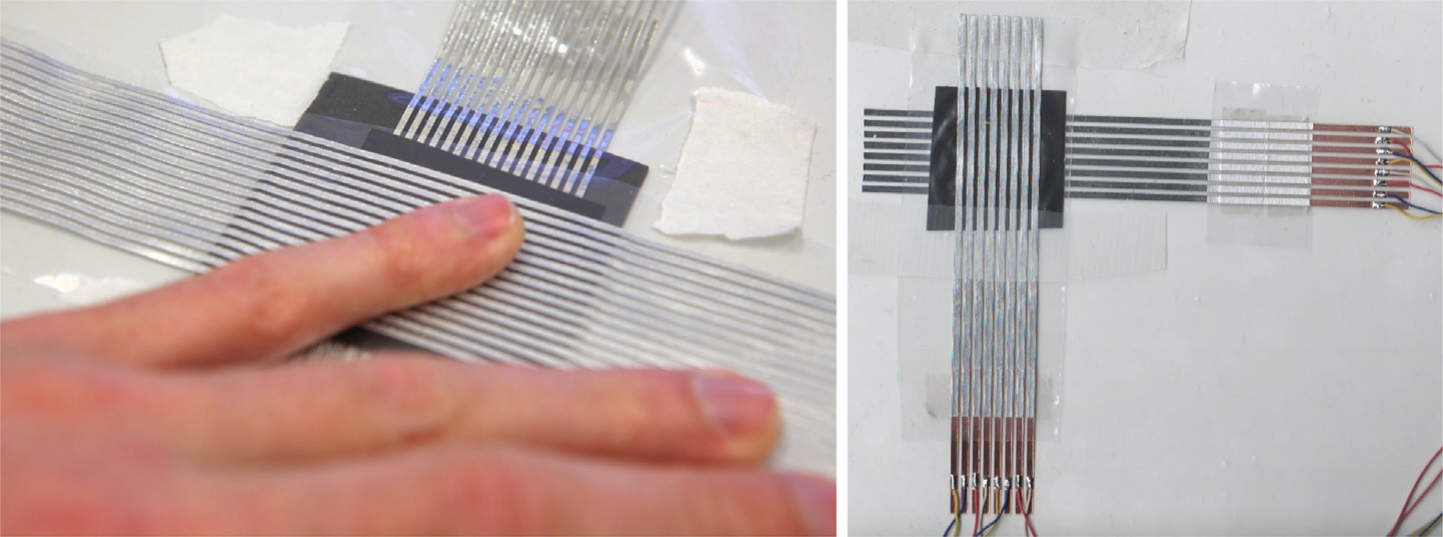
Examples for alternative sensor resolutions and connection methods.

The same procedure can also be used to produce sensors with a resolution higher than 6×6 taxels, as demonstrated in Fig. 9. Arrays with 16×16 taxels (left) and 8×8 taxels (right) are shown. Measurement results of a sensor array with 16×16 taxels are presented in Fig. 14. Then, however, an alternative sensor module with more ADC–channels is required. Further, the right image in Fig. 9 also shows an alternative connection method between the sensor and the sensor module. Strips of copper are glued to the aluminum traces using conductive tape. In contrast to the aluminum traces, it is possible to solder cables to the copper strips. This allows setups without an immediate connection between the sensor traces and a sensor module.

### 5.3 Sensor module setup

**Fig. 10:**
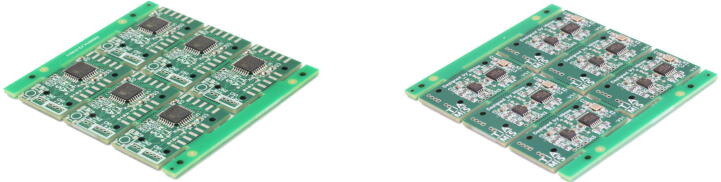
Populated Tacduino sensor module PCB from the top **(left)** and bottom **(right).**.

As described in Section 2.2, the sensor module mainly combines a microcontroller with transimpedance amplifiers for sensor signal amplification and decoupling. A miniaturized implementation of the sensor module with applications in various setups was designed. We outsourced most of the production of the sensor module by ordering the PCBs from a fabricator who also populated one of the two sides. The other side (with the microcontroller) is deliberately designed to be easier to solder by hand. To reduce cost, the panelization feature was used to fit six sensor modules onto a single board (see Fig. 10). The individual modules can be broken apart when needed. Connectors for plugs are provided, which makes maintenance easier. However, the usage of these is often not practical due to space restrictions. For such cases, solder pads are provided to directly solder leads to. This also allows for cutting off a part of the PCB, further reducing its size, which is described in the following section.

### 5.4 Main module setup

Before flashing the code to the main module, the main_controller.ino file needs to be adapted to the current setup of the system. Set the definitions for BLUETOOTH and SERIAL to false or true as required. Note that it is possible to use both simultaneously, but it will reduce the transmission rate. Afterwards, the list i2c_addresses needs to be filled with the I^2^C addresses of all sensors in the system. Then, the sensor_numbers have to be defined by a list of the same length as the i2c_addresses. Here, it is possible to assign sensor numbers to each sensor (in the same order as the i2c_addresses), to allow for edge cases such as two sensor modules with the same I^2^C address connected to two main modules in the same system. However, to prevent confusion, we recommend to use similar sensor_numbers and i2c_addresses. In the same manner, enter the values for the sensor byte counts. The sensor byte count *C* can be calculated using the following formula: C=1+⌈n*(k/8)⌉ with *n* being the number of taxels and *k* the number of bits per sensor reading. Because the ADCs of the ATmega328p have a precision of 10 bit, we use k=10 by default. So for our sensor arrays with 6×6 taxels, we get C=1+⌈36*(10/8)⌉=46. The one byte added at the beginning of the formula is used to include the sensor ID in its data package. After adapting the file, it can be flashed onto the Arduino Nano clone using the Arduino IDE.

### 5.5 Interconnection of modules

**Fig. 11:**
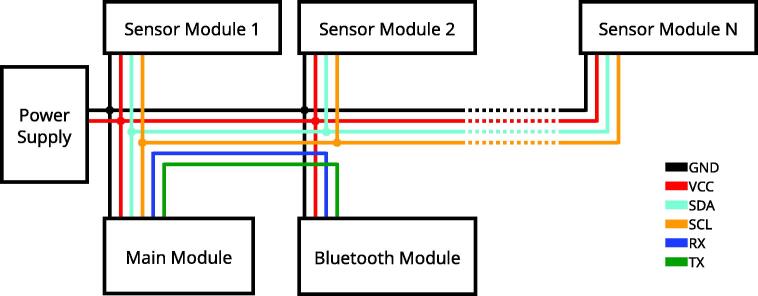
Wiring diagram of the full system in wireless configuration.

To complete the system setup, all components need to be linked up. Fig. 11 shows a wiring diagram for a full system configured for wireless data transmission. All sensor modules are connected to a power supply and the I^2^C bus (by SDA and SCL). The main module is interfacing between the I^2^C bus and the Bluetooth module. When using an Arduino Nano as main module, use pins 10 and 11 for the RX and TX connections to the Bluetooth module and pins A4 and A5 for the SDA and SCL connections to the I^2^C bus. When a wired data transmission via the USB port of the main module is used, the host PC acts as the power supply, the Bluetooth module is not used anymore. Additional data wires (RX and TX) are not necessary because the serial data stream is converted to USB on the Arduino board itself. A Robotiq 3-Finger Gripper equipped with three fingertips, the production of which we detailed, and a main module in the wireless configuration, are shown in Fig. 1b.

## 6 Operation instructions

For the operation of the sensor arrays, we supply Python files that are able to read the sensor data both in the ROS ecosystem and outside of it on Linux systems. To use them, a configuration file describing the sensor system needs to be set up. Listing 1 shows an exemplary configuration file of the sensor driver for the host PC.

Listing 1. Exemplary host PC configuration file.

**Table:** 

First, general settings for the communication with the main module and the ROS system are set. The scrips support either a serial or a Bluetooth connection. The Bluetooth connection can be activated in line 2, while the serial connection is used when the serial connection is set to False.

Then, for each sensor, the individual settings need to be defined. First, a unique ID is set (the one which was assigned to the sensor’s address in the main module code in Section 5.4). Second, the sensor size is entered by giving width and height. This information is used to compute the size of the data packages received and added to sensor data messages for visualization purposes. Thus, when a custom shape is used, use a layout such as 1×(A*D) with *A* being the number of analog channels and *D* being the number of digital channels of the sensor module used. As mentioned in Section 5.4, the value size is normally 10 bits, so if an ATmega328p is used on the sensor module with its internal ADCs and the sensor module code is left as is, this should be left at 10. Finally, the ROS TF frame of the sensor link can be set. This value is used in the header of the sensor data message and can be set freely.

When using the standalone driver, we recommend fetching sensor data by creating a SensorReader-object and either using the receive_one-method or by setting continuous_run to True and passing a callback function to the constructor, which will be called when sensor data is received. The constructor of the SensorReader-class requires the configuration file parsed into a dictionary as a parameter. We recommend using the PyYaml package for this.

In the ROS environment, the code can be built as a Catkin package. After building the package, the configuration file is loaded and started using the tactile_sensor.launch file. While the node is running, other nodes can subscribe to the published sensor data, which will be sent in the form of a TactileSensorArrayData message.

## 7 Validation and characterization

We demonstrate the wide applicability of our hardware by integrating it into diverse robotic setups, as shown in Fig. 12. The palm sensor for the Shadow Dexterous Hand features a complicated non-rectangular shape of the sensor matrix, required to keep the full motion range of the thumb and little finger of the hand. On its fingertips, the hand is equipped with BioTac sensors which can be used for precision grasps. The arrays of tactile sensors augment the sensing capabilities for power grasps. For the proximal and medial phalanges, a more flexible sensor such as the one presented by Ruppel et al. is required [20]. The instrumented can demonstrates a curved outer sensor surface with 16×32 taxels. Further, we analyze the sensor characteristics by measuring its response to known input stimuli. The data shown here extends the analysis performed for the original paper [1].

Due to the high number of variables in the sensor design, we cannot provide generalized specifications for the sensor as they depend strongly on the type of piezoresistive foil, but also on the value of the feedback resistors, the width of the aluminum traces, the thickness of the silicone layer, and environmental factors such as room temperature.

**Fig. 13:**
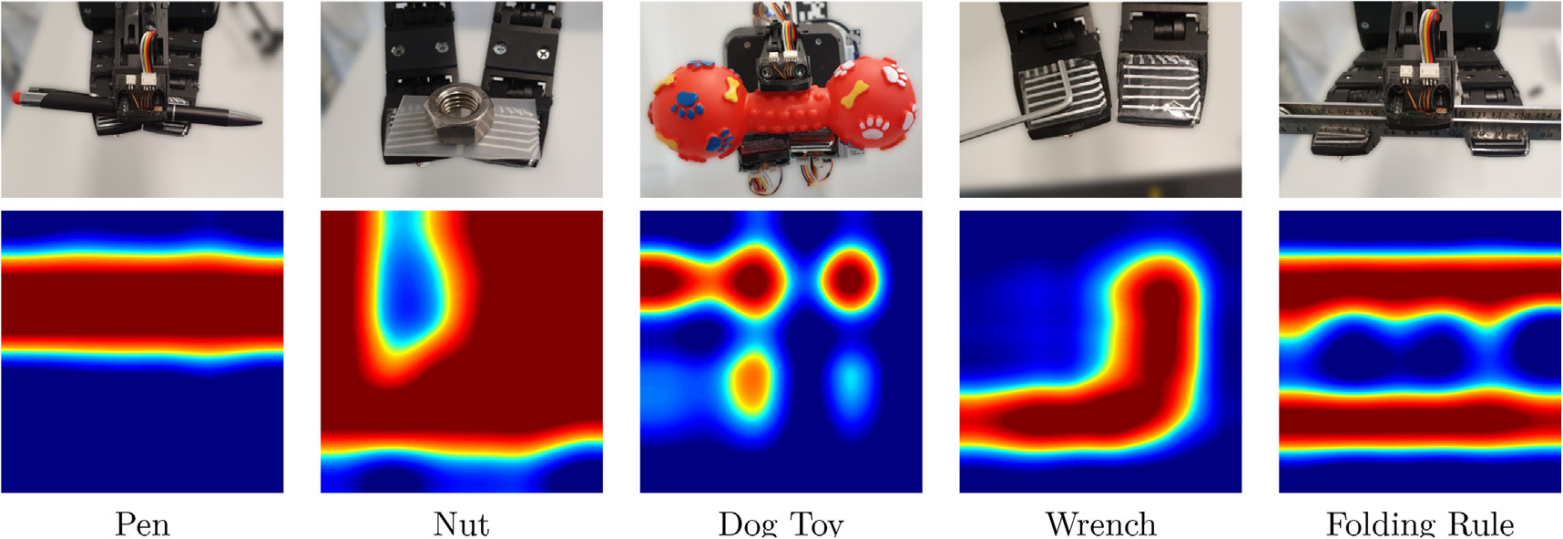
Diverse stimuli and a heatmap visualization of the sensor data acquired at the fingertips of the gripper. The visualized recordings were taken while the gripper was closed. The data was taken from the upper finger for the pen, nut, and dog toy, from the left finger for the wrench, and from the right finger for the folding rule. [1].

**Fig. 14:**
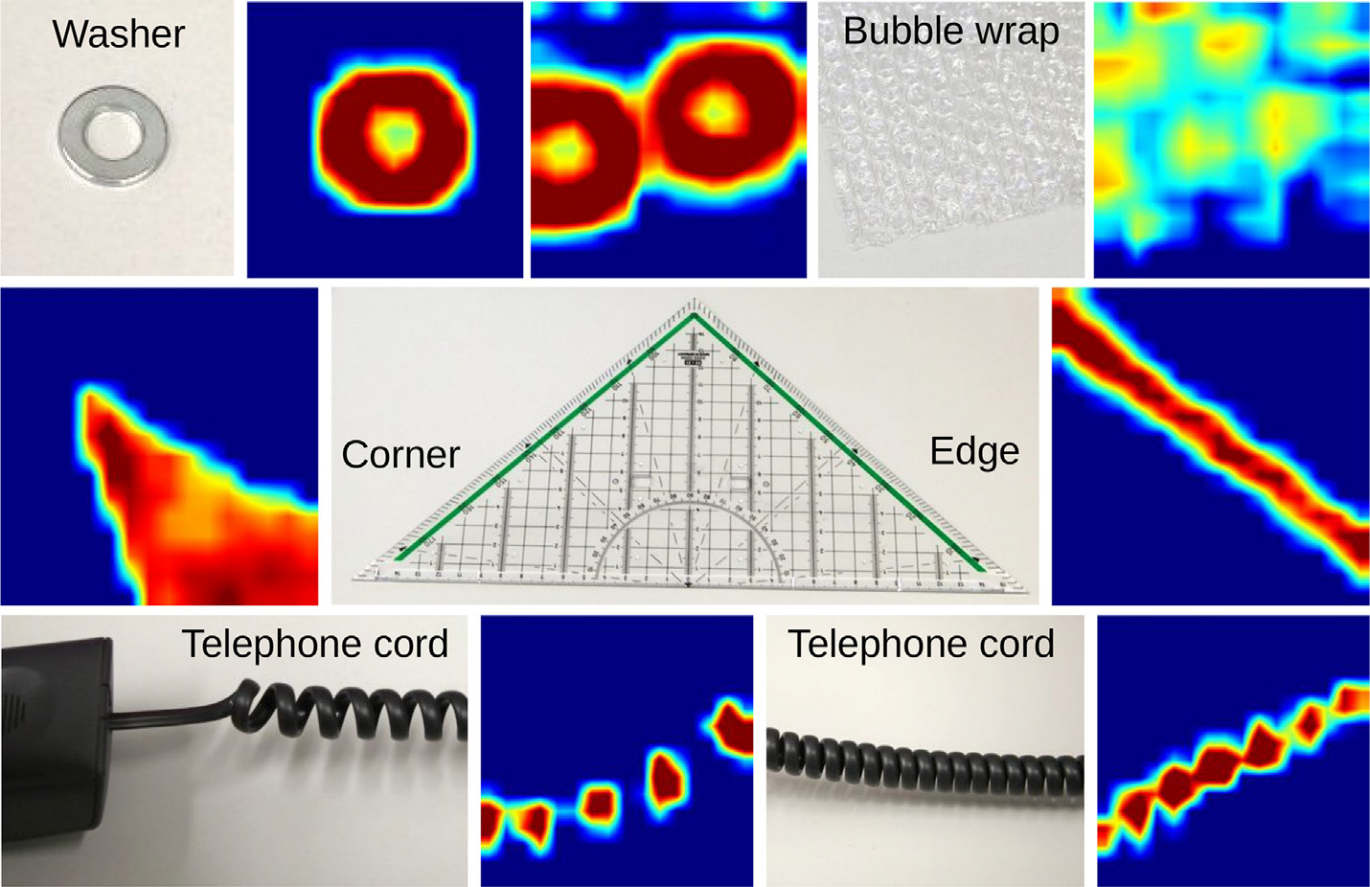
Response of a low-cost sensor matrix to different objects and textures. Washers (top-left), bubble wrap (top-right), corner and edge of a triangle ruler (middle), telephone cords (bottom).

To give a general impression of the sensor’s response to stimuli, we show visualizations of the sensor’s measurements taken when interacting with commonly found objects in Fig. 13. The surface shapes of the objects are well represented and clearly recognizable. We performed similar experiments using an array with 16×16 taxels, the results of which are shown in Fig. 14. The measurements are visualized with a color map from dark blue, via cyan, green, and yellow to dark red. Dark blue represents low measurement values (no contact) and dark red indicates the highest measurable pressure (saturation).

**Fig. 15:**
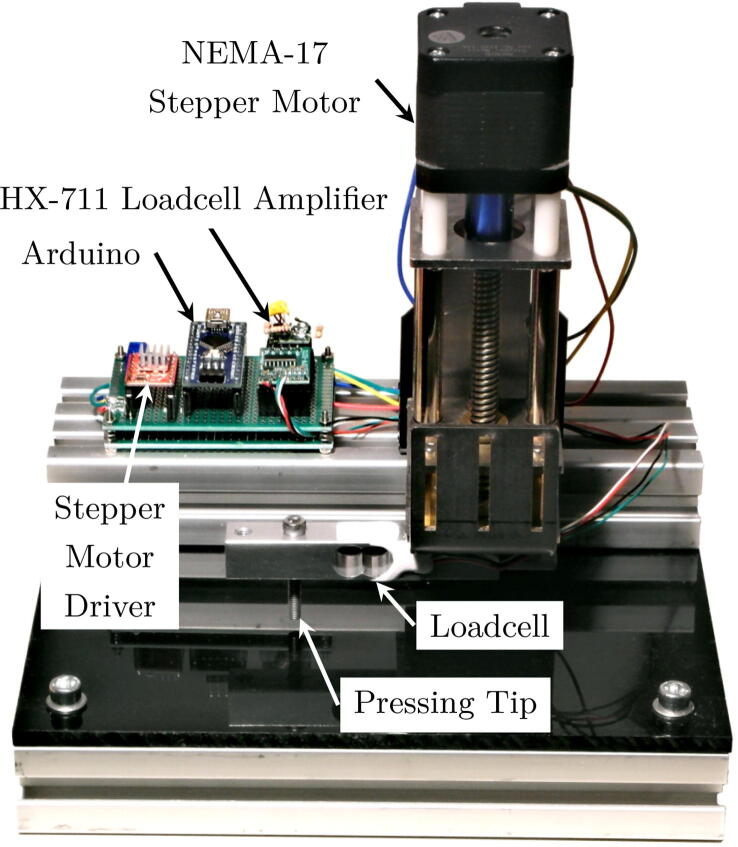
Prototype calibration jig to verify and characterize sensor responses. The sensor is placed on the jig with one sensor cell below the pressing tip, which is then lowered onto the sensor matrix via the z-spindle. Driven by a stepper motor, the setup provides very fine steps and high accuracy, while the applied force is measured by the pre-calibrated loadcell.

For detailed characterization and analysis of the sensor’s response, we built a calibration jig as shown in Fig. 15. It uses a stepper motor to press a pin mounted on a loadcell onto a taxel of the sensor. Using the setup, we are able to stably apply a force to the taxel and compare the known stimuli to the sensor readouts. We performed the measurements visualized in Fig. 16 by pressing on a single taxel of our sensor array in six different configurations. 10 kΩ and 1 kΩ feedback resistors were evaluated each without a silicone layer and with a thin (1.5 mm) and a thick (2.5 mm) layer of silicone on the sensor surface. Note the difference in scale of the *x*-axis between the upper and lower row of the plots. With a 10 kΩ feedback resistor, saturation occurs at an applied force over about 4–5 N. If a wider measurement range is required, we recommend using a smaller feedback resistor. The results demonstrate that the sensors can reliably detect and measure typical grasping forces.

## Ethics statements

Neither animals nor human subjects were involved in this work.

## CRediT author statement

**Niklas Fiedler**: Writing - Original Draft, Methodology, Investigation, Software **Philipp Ruppel**: Conceptualization, Methodology, Investigation, Writing - Review & Editing **Yannick Jonetzko**: Writing - Review & Editing, Resources, Visualization **Norman Hendrich**: Writing - Review & Editing **Jianwei Zhang**: Supervision, Funding acquisition.

## Declaration of Competing Interest

The authors declare that they have no known competing financial interests or personal relationships that could have appeared to influence the work reported in this paper.

## Figures and Tables

**Table:** 

ADC	Analog-to-digital converter
CAD	Computer-aided design
I^2^C	Inter-integrated circuit
PCB	Printed Circuit Board
ROS	Robot Operating System
SCL	Serial Clock
SDA	Serial Data
SVG	Scalable Vector Graphics
TDFN	Thin Dual Flat No Leads
TF	Transform Frames
TQFP	Quad Flat Package
TSSOP	Thin Shrink Small Outline Package
USB	Universal Serial Bus

## Appendix A

Acronyms.
